# Myofiber HLA-DR expression is a distinctive biomarker for antisynthetase-associated myopathy

**DOI:** 10.1186/s40478-014-0154-2

**Published:** 2014-10-23

**Authors:** Jessie Aouizerate, Marie De Antonio, Guillaume Bassez, Romain K Gherardi, Francis Berenbaum, Loïc Guillevin, Alice Berezne, Dominique Valeyre, Thierry Maisonobe, Odile Dubourg, Anne Cosnes, Olivier Benveniste, François Jérôme Authier

**Affiliations:** Reference Center for Neuromuscular Diseases, Hôpital Henri Mondor, APHP, Créteil, France; INSERM U955- Team 10; Paris Est-Creteil University, Créteil, France; INSERM U1138, Paris Descartes University, Paris, France; Rhumatology, Hôpital Saint Antoine, APHP, Paris, France; Internal Medicine, Hôpital Cochin, APHP, Paris, France; Pneumology, Hôpital Avicenne, APHP, Bobigny, France; Neuropathology, Hôpital Pitié Salpétriêre, APHP, Paris, France; Dermatology, Hopital Henri Mondor, APHP, Créteil, France; Internal Medicine, Hopital Pitie-Salpetriere, APHP, Paris, France; Département de Pathologie, Hopital Henri Mondor, 51, Av. du Maréchal de Lattre de Tassigny, 94010 Creteil, France

**Keywords:** HLA-DR, Inflammatory myopathy, Dermatomyositis, Anti-synthetase, Complement

## Abstract

**Objectives:**

To assess the value of major histocompatibility complex (MHC) class II antigen (HLA-DR) expression to distinguish anti-synthetase myopathy (ASM) from dermatomyositis (DM).

**Methods:**

Muscle biopsies from patients with ASM (n = 33), DM without anti-synthetase antibodies (ASAb) (n = 17), and normal muscle biopsy (n = 10) were first reviewed. ASAb included anti-Jo1 (26/33), anti-PL12 (4/33), anti-PL7 (2/33), and anti-EJ (1/33). Immunohistochemistry was performed for MHC-I/HLA-ABC, MHC-II/HLA-DR, membrane attack complex (C5b-9), neural cell adhesion molecule (NCAM)/CD56 expression, and inflammatory cell subsets. Twenty-four ASM and 12 DM patients from another center were added for HLA-DR evaluation.

**Results:**

Ubiquitous myofiber HLA-ABC expression was equally observed in ASM and DM (93.9% vs 100%, NS). In contrast, myofiber HLA-DR expression was found in 27/33 (81.8%) ASM (anti-Jo1: 23/26, 88.5%; others: 5/7, 71.4%) vs 4/17 (23.5%) DM patients (p < 0.001). HLA-DR was perifascicular in ASM, a pattern not observed in DM. In addition, C5b-9 deposition was observed on sarcolemma of non-necrotic perifascicular fibers in ASM, while, in DM, C5b-9was mainly detected in endomysial capillaries. CD8 cells were more abundant in ASM than in DM (p < 0.05), and electively located in perimysium or in perifascular endomysium. HLA-DR expression correlated positively with the CD8+ cells infiltrates. Strictly similar observations were made in the confirmatory study.

**Conclusion:**

ASM is characterized by strong myofiber MHC-II/HLA-DR expression with a unique perifascicular pattern, not described so far. HLA-DR detection must be included for routine myopathological diagnosis of inflammatory/dysimmune myopathies. HLA-DR expression in ASM may indicate a specific immune mechanism, possibly involving IFNγ.

**Electronic supplementary material:**

The online version of this article (doi:10.1186/s40478-014-0154-2) contains supplementary material, which is available to authorized users.

## Introduction

Idiopathic inflammatory and dysimmune myopathies (IDM) form a group of acquired conditions characterized by immune-mediated injuries of muscle tissue. Most of them favorably respond to immunomodulatory therapies making necessary their accurate identification at early stages of evolution. On the grounds of histopathological criteria, IDM are usually divided in three main groups [1,2]: (i) polymyositis (PM), (ii) dermatomyositis (DM), and (iii) autoimmune necrotizing myopathies (AINM). From an immunological view, PM are characterized by a cell-mediated autoimmune response directed towards myofibers, as assessed by abnormal ubiquitous MHC class I/HLA-ABC myofiber re-expression and endomysial CD8 T-cells surrounding and invading non-necrotic fibers. This feature is also observed in inclusions body myositis (IBM), which in turn differs from PM by the presence of chronic myopathic changes, rimmed vacuoles, aggregates and mitochondrial abnormalities. DM is a vasculopathic myopathy with perimysial vascular inflammation, myofiber ischemic lesions, and endomysial microangiopathy with complement activation [3]. AINMs are defined by random myofiber necrosis with complement activation and minimal inflammation [2,4].

The identification of myositis-specific autoantibodies (MSA) helped to identify peculiar subsets of IDM patients with specific clinical and prognostic implications [1]. This is typically the case of ‘anti-synthetase syndrome’ (AS), a systemic disease characterized by inflammatory myopathy, interstitial lung disease, joint involvement, and skin lesions. This syndrome is defined by the presence of circulating autoantibodies directed against tRNA-synthetase, most often histidyl- (anti-Jo-1), and less frequently threonyl (anti-PL-7), alanyl (anti-PL-12), isoleucyl (OJ), glycyl (EJ), asparaginyl (KS), phenylalanyl (Zo) and tyrosyl (Ha) [5,6]. Anti-Jo-1 antibodies are found in 25-30% of patients with inflammatory myopathy and other anti-synthetase antibodies (ASAb) in 1–5% [7–9]. Whether ASAb-associated myopathy (ASM) is an independent disease among IDM or corresponds to a known entity like PM, DM or AINM is debated [1,10,11]. The reappraisal of myopathological findings in patients with anti-Jo-1 syndrome displayed peculiar features including (i) fragmentation of perimysial connective, (ii) septal inflammation (predominantly macrophages), and (iii) myofiber lesions restricted to perifascicular area [12], these abnormality forming a pattern termed ‘immune myopathy with perimysial pathology’ (IMPP) by A. Pestronk [10]. Notably however, muscular involvement in ASM resembles DM and ASAb can be detected in some patients with definite DM [13]. In both conditions, myofiber alterations predominate in perifascicular areas, and in addition, perimysial connective tissue fragmentation is unexceptionally observed in DM [1,10]. On the other hand, alkaline phosphatase staining of perimysium and adjacent endomysium seems is very unusual in DM while regularly observed in ASM [10]. Finally, DM is characterized by endomysial microangiopathy [3], a feature not usually observed in ASM [10], although occasionally reported [14].

Current diagnostic approaches of IDM require the histopathological evaluation of immunological parameters including lymphocyte phenotype, myofiber MHC-I expression and complement activation (C5b9 formation). Myofiber MHC-I/HLA-ABC expression has been extensively studied and was shown to be a very sensitive but poorly sensitive marker of inflammatory myopathy [15–17]. In contrast, only a few works evaluated the possible significance of MHC-II/HLA-DR myofiber expression [17–21]. Unlike MHC-I, there is no constitutive expression of MHC-II/HLA-DR molecules by myogenic cells, *in vivo* or *in vitro* [22,23]. Myofiber HLA-DR expression was ascribed to the presence of interferon (IFN)γ in the microenvironment of myofibers [19,23,24]. Before delineation of ASM, HLA-DR immunostaining was proposed as a marker of inflammatory myopathy [18,25], but further studies failed to attribute a diagnostic value to the finding [26]. In the present study, we investigated muscle HLA-DR expression in ASM and ASAb-negative DM, and compared it to the expression of other histopathological biomarkers. As opposed to ASAb-negative DM, ASM was characterized by strong myofiber HLA-DR expression, electively localized in perifascicular areas, and typically associated with C5b9 deposition on sarcolemma of non necrotic fibers, and not on capillaries. This characteristic pattern was confirmed in a second series of patients from another center pointing out ASM as a distinct type of IDM.

## Materials and methods

### Patients

We retrospectively studied 50 adult patients (age > 18 years) who underwent muscle biopsy for diagnostic purposes in our institution (Henri Mondor University Hospital) between January 2004 and January 2013. All patients were suspected to have inflammatory myopathy and had comprehensive ASAb testing including anti-Jo1, anti-PL7, anti-PL12, anti-EJ, and anti-OJ autoantibodies. Patients were divided in two groups: Group 1: ASM patients characterized by positive detection of ASAb (ASM, n = 33); Group 2: ASAb-negative DM patients (n = 17). DM diagnosis was based on ENMC 2003 criteria [1]. Ten patients with fibromyalgia and normal muscle biopsy were used as controls. In accordance with current French legislation; all patients gave written consent (Approval #12-009 at CPP Ile-de-France IX).

All controls had normal physical examination, normal CK level, negative ASAb testing. ASM patients included 18 males and 15 females. Age at muscle biopsy ranged from 26 to 74 years (mean 52.8). Myalgias were present in 26/26 patients (100%) and muscle weakness in 18/26 (69.2%). Extra-muscular features included: skin changes (heliotrope rash: 8/29, 27.6%; Gottron’s signs: 4/29, 13.8%; periungual changes: 2/29, 6.7%; mechanic hands: 6/29, 20.7%); Raynaud’s syndrome (7/29, 30.4%); pulmonary involvement (21/26, 80.8%); and rheumatic signs (joint pain or arthritis: 24/26, 92.3%). Initial diagnosis was classic DM in 2 anti-Jo1 patients. Creatine phosphokinase (CK) serum level was increased in 17/19 (mean 2198 ui/l, range: 96–10000; normal values: 40–225). The mean delay between the onset of symptoms and the first muscle biopsy was 10.7 months (range 0–24). In all ASM patients, serum displayed positive cytoplasmic fluorescence on Hep2 cells due to the presence of circulation of ASAb (starry sky pattern). ASAb were anti-Jo1 (26/33), anti-PL12 (4/33), anti-PL7 (2/33), and anti-EJ (1/33). Two ASM patients also had anti-Ro52 antibodies.

DM patients included 6 males and 11 females. Age at muscle biopsy ranged from 18 to 84 years (mean 59.2). 16/17 (94.1%) patients had overt skin changes before biopsy. Myalgias were present in 15/17 (88.2%) and muscle weakness in 12/17 (70.6%). Extra-muscular features included pulmonary involvement (4/17; 23.5%), and rheumatologic signs (joint pain or arthritis, 6/16; 37.5%). CK serum level was found increased in 8/11 (mean 2388ui/l, range: 47–12209; normal: 40–225). In DM patients, antinuclear antibodies were positive in 11/17 (speckled pattern 8/17; homogen pattern 3/17). Associated malignancy was noted in 9/17 (52.9%) patients. The mean delay between the onset of symptoms and the muscle biopsy was 3 months (range 0–13). Pulmonary involvement was more frequent in ASM (80.8% in ASM vs 23.5% in DM, p < 0.001) and associated malignancy more frequent in DM (none in ASM vs 52.9% in DM).

### Myopathological study

All patients had open deltoid or quadriceps muscle biopsy. Muscle specimens were conventionally processed [3,27]. One muscle sample was flash-frozen in isopentane cooled by liquid nitrogen for histoenzymology and immunohistochemistry, another fixed in formaldehyde fixative for routine histology, and the last processed for electron microscopy. Unfixed 7 μm cryosections were routinely stained with hematein-eosin (HE), Gomori or Masson trichrome, PAS, Sudan black, and histoenzymological reactions for NADH-TR and cytochrome-C-oxidase (Cox).Myosinolysis, microinfarctus and perifascicular atrophy were assessed as previously proposed [28,29]. Immunoperoxidase investigations were carried out on frozen 7 μm sections using a Bond-III automaton (Leica, Nanterre, France) to detectHLA-ABC, HLA-DR, membrane attack complex (MAC/C5b-9), regenerating myofibers (CD56/neural cell adhesion molecules [NCAM]), macrophages (CD68), and other leukocyte subsets CD3, CD4, CD8 CD20. Detailed characteristics of primary antibodies are in Additional file 1: Table S1. Morphological analyses were carried out on Axioskop® 2 light microscope (Zeiss) and digital image capture using Coolscope® (Nikon) device.

### Quantitative assessment of immunopathological parameters

The severity of myonecrosis process was estimated by determining the percentage of NCAM-positive regenerating fibers. Myofiber expression of HLA-ABC and HLA-DR was defined by sarcolemmal staining associated or not with sarcoplasmic staining. The percentage of HLA-ABC-positive fibers was determined in three representative fascicles. Quantitative evaluation of HLA-DR expression was performed in the most affected fascicle and included (i) the percentage of positive fibers in the whole fascicle, and (ii) the percentage of contiguous positive fibers among the external rim of perifascicular fibers. C5b-9 decoration of myofibers was considered in case of sarcolemmal labeling. In contrast, fibers with marked C5b-9 intracellular immunoreactivities were considered necrotic and not taken into account. Quantitative evaluation of myofibers C5b-9 labeling was performed in the most affected fascicle and included (i) the percentage of positive fibers in the whole fascicle, and (ii) the percentage of positive fibers among the external rim of perifascicular fibers. The amounts of CD3+, CD4+, CD8+, CD20+ and CD68+ cells were scored following the score recently assessed for juvenile DM [29].

### Statistical analysis

Statistical analysis was done using R software (www.R-project.org). Qualitative criteria were compared using Fisher’s exact test. In case of non normal distribution, quantitative variables were compared using Mann Whitney U test. To compare more than two groups, the Kruskal-Wallis test was used. Dunn post-hoc test was used for pairwise comparisons. All p-values were two-tailed and statistical significance is defined as p < 0.05.

## Results

### Myopathological evaluation

**Figure 1:**
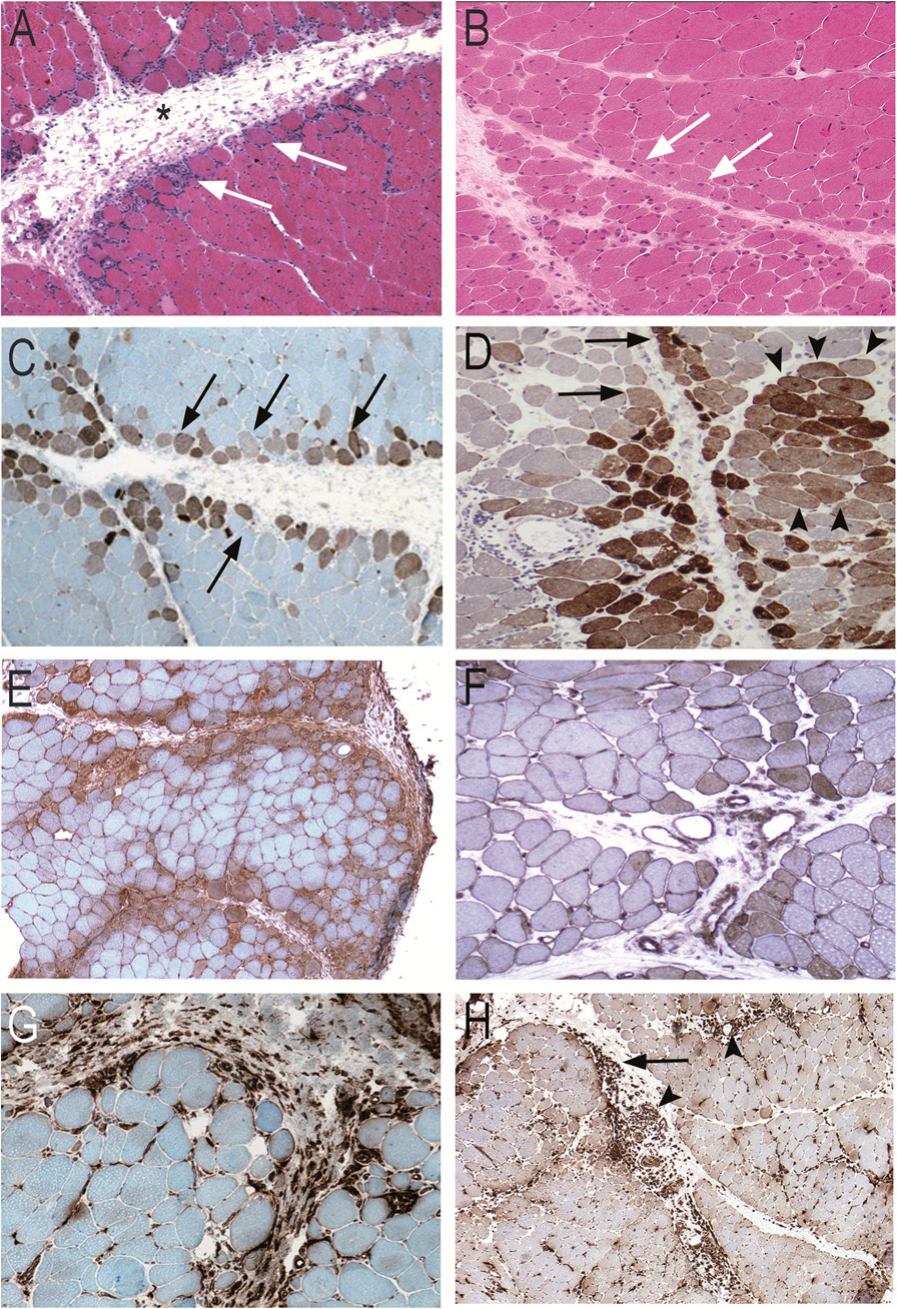
**Myopathological features in ASM (A, C, E, G) and DM (B, D, F, H). A**: Myofiber injuries in ASM predominate in perifascicular areas (arrows) with the rounded atrophic appearance of regenerating myofibers; inflammatory infiltrates are mainly observed in perimysium and perifascicular endomysium and are associated with fragmentation of perimysium (asterisk). **B**: Perifascicular atrophy in DM characterized bymyofibers in peripheral regions of fascicles with a cross-sectional area substantially lower than in the rest of the fascicle(arrows). **C**: Myofiber NCAM expression (arrows) typically restricted to perifascular layers in ASM. **D**: In DM, NCAM-expressing myofibers are both perifascular (arrows) or grouped in foci (arrowheads). **E**, **F**: Diffuse myofiber reexpression of HLA-ABC in ASM **(E)** and DM **(F)**, with marked perifascicular reinforcement in ASM **(E). G**, **H**: Macrophages in perimysium in the vicinity of fascicles (arrows) in ASM **(G)**, and in perimysium (arrows) and perivascular areas (arrowhead) in DM **(H)**. Frozen sections; hematoxylin-eosin **(A, B)** and immunoperoxidase technique for NCAM (C, D), HLA-ABC **(E, F)** and CD68 **(G, H)**.

**Figure 2:**
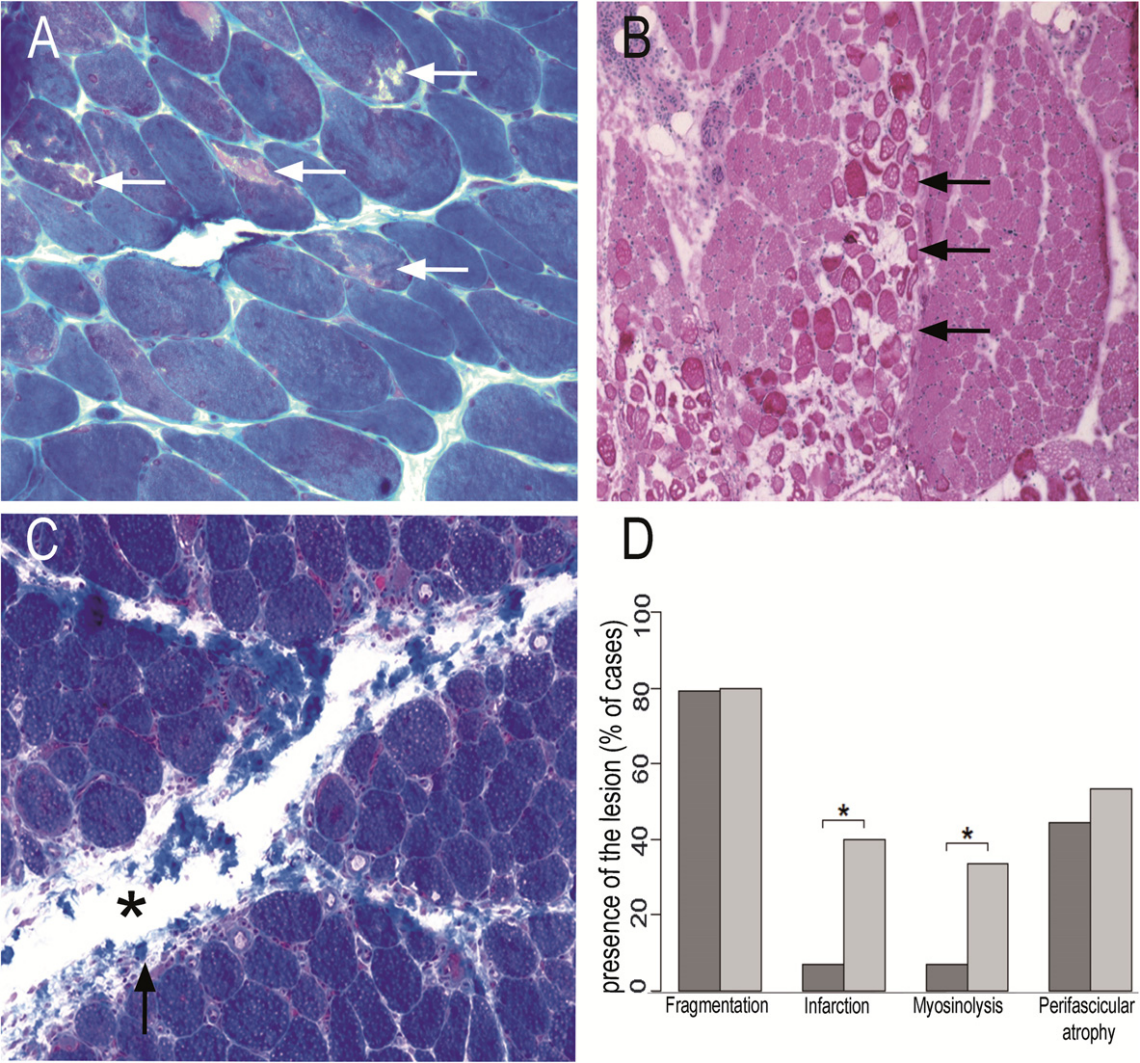
**Comparison of myopathological lesions in DM (A, B) and ASM(C). A**: Myosinolysis defined by the presence of punched-out vacuoles (arrows) or myofibrillar rarefaction areas, corresponding to foci of myosin filament proteolysis. **B**: Microinfarcts defined by numerous contiguous fibres within a fascicle, at the same stage of necrosis or regeneration. **C**: Perimysial fragmentation defined by focal clear areas (asterisk) and spumous appearance of adjacent areas (arrowhead). **D**: Percentages of cases with presence of the lesion (perimysial fragmentation, microinfarcts, vacuoles, and perifascicular atrophy) in ASM and DM. (*) indicates p < 0.05.Frozen sections **(A-C)**; hematoxylin-eosin **(A, B)** and Masson trichrome **(C)**.

Perimysial connective tissue fragmentation and perifascicular atrophy were present in both AS and DM (79.3 vs 80.0% (NS) and 44.4 vs 53.3% (NS) respectively) (Figures 1A-B; 2C). In contrast, microinfarcts were observed in only 6.9% ASM *vs* 40.0% of DM patients (p < 0.05). Ischaemic myosinolysis and punched-out vacuoles were observed in 6.9% AS and 33.3% in DM (p < 0.05) (Figure 2A-B, D)

### Myofiber HLA-ABC and HLA-DR expression

**Figure 3:**
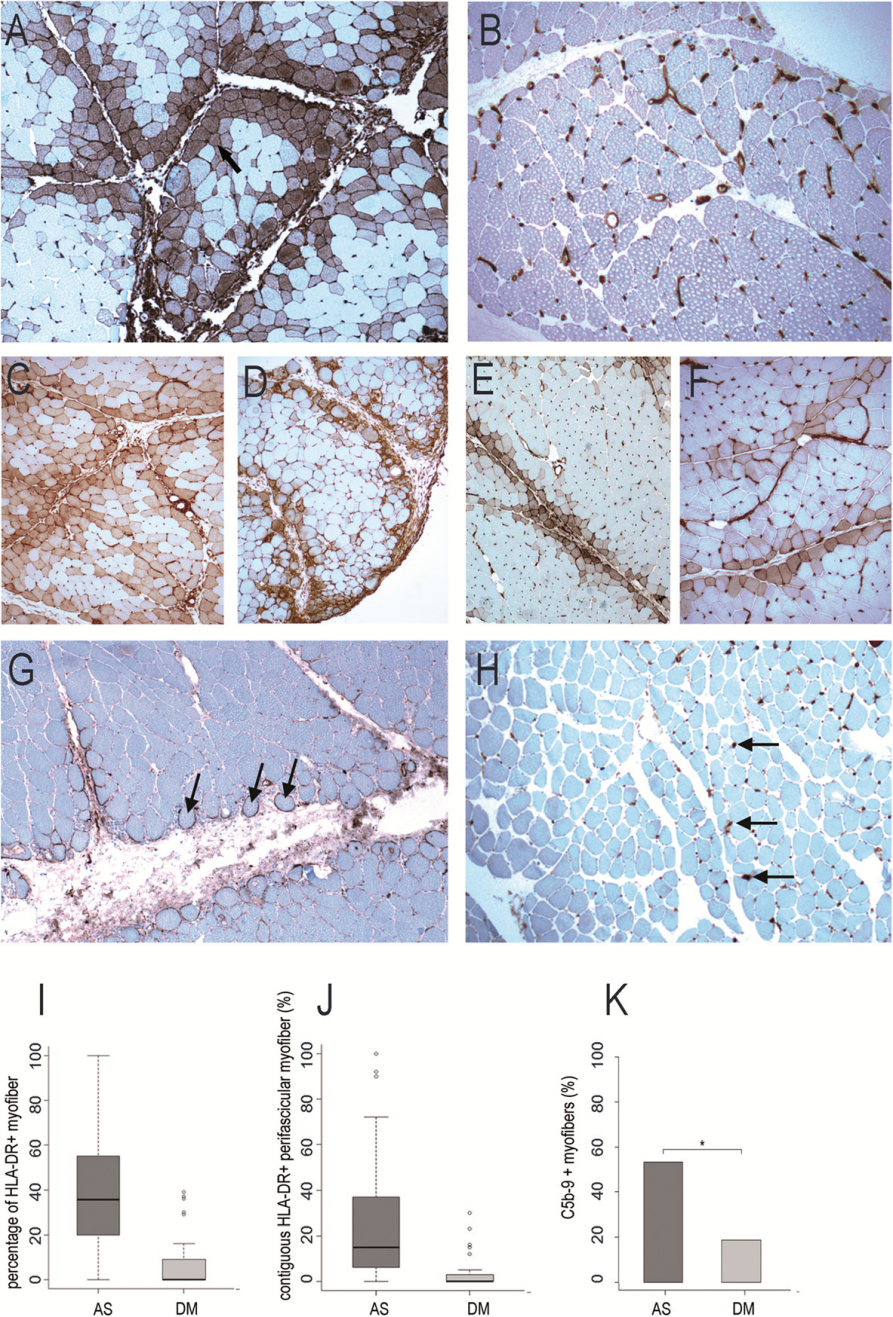
**HLA-DR expression and C5b-9 deposition in ASM and DM. A**-**F**: Regular myofiber HLA-DR expression in perifascicular areas in ASM **(A, C-F)** contrasting with the lack or very few number of positive myofibers in DM **(B)**; positive staining of endomysial vessels (arrows) is physiological **(C)**.** G, H**: C5b-9 deposition observed at the surface of perifascicular myofibers in ASM **(G)** and in endomysial vessel walls (arrows) in DM **(H)**. **I**,**J**: Percentage of HLA-DR-positive myofibers **(I)** and of contiguous HLA-DR-positive perifascicular myofibers **(J)** in ASM and DM. **K**: Percentages of cases with presence of C5b-9 deposition at the surface of non-necrotic myofibers in ASM and DM. (*) indicates p < 0.05.Frozen sections **(A-H)**; immunoperoxidase technique for HLA-DR **(A-F)** and C5b-9 **(G, H)**.

HLA-DR myofiber expression was found in 48/57 (82.8%) patients with ASM (anti-Jo1: 44/50, 88%; others: 5/7, 71.4%) and in 8/29 (27.6%) patients with DM (p < 0.001, Fisher’s test). No myofiber expression of HLA-DR was found in control muscles. The mean percentage of positive fibers in the most positive fascicle was 39.1% in ASM and 7.5% in DM (DM vs ASM: p < 0.001; DM vs anti-Jo1: p < 0.001; Fisher’s test) (Figure 3A-F, I,J). A striking feature of ASM was the perifascicular distribution of HLA-DR expression (Figure 3A, C-F).This pattern was observed in all ASM patients who had HLA-DR + myofibers. The mean percentage of contiguous HLA-DR + perifascicular fibers was 26.5 in ASM (35.1 in anti-Jo1 syndrome) versus 2.2 in DM (p < 0.05; [DM vs anti-Jo1: p < 0.001]) (Figure 3J). These results indicate that ASM muscle biopsy typically shows strong myofiber expression of HLA-DR, with perifascicular pattern.

### Complement activation pattern

C5b-9 deposits were observed in both ASM and DM muscles but with strikingly distinctive patterns. All DM cases displayed microvascular C5b-9 immunoreactivities, which were usually clustered in small foci of positive capillaries (Figure 3H). In contrast, in ASM, C5b-9 deposition was detected at the surface of non-necrotic fibers (17/32 cases; 53.1%) (Figure 3G) and also in capillaries (24/32; 75%). In DM C5b-9 myofiber immunoreactivities were very few (3/16 cases; 18.7%) (ASM*vs* DM, p = 0,03) (Figure 3K). The most distinctive feature was the electively perifascicular distribution of C5b-9 sarcolemmal distribution in ASM (40.6% in ASM vs 6.3% in DM, p = 0,02) (Figure 3G, K).

### Other immunostaining

Myofiber expression of HLA-ABC was indistinctively observed in ASM patients (31/33, 93.9%; anti-Jo1: 26/26, 100%; others: 5/7, 71.4%) and 17/17 DM patients (100%) (NS; Fisher’s test), and not detected in control muscles. HLA-ABC expression was ubiquitous or almost ubiquitous with perifascicular reinforcement in both groups (Figure 1E, F). Regenerative NCAM + fibers were found in similar proportion in both groups: 15.4% in ASM (16.4% in anti-Jo1; 11.7% in others) and 19.1% in DM (NS; Fisher’s test) (Figure 1C, D).Macrophages were observed in perimysium close to fascicles in ASM (Figure 1G), and in perimysium and perivascular areas in DM (Figure 1H).

**Figure 4:**
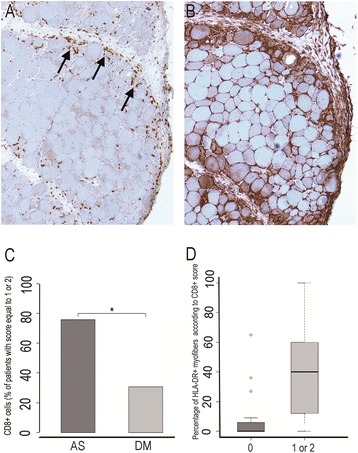
**CD8+ T-cell infiltration and myofiber HLA-DR expression. A**, **B**: Comparison of CD8+ T-cells infiltration and myofiber HLA-DR expression in a same case of ASM. CD8+ T-cells (arrows) are detected in perifascicular endomysium and adjacent perimysium **(A)**, in the areas corresponding to those of the strongest myofiber HLA-DR expression **(B)**. Frozen section; immunoperoxidase technique for CD8 and HLA-DR. **C**: Percentage of patients with a CD8+ score equal to 1 or 2, in ASM and DM; (*) indicates p < 0.05. **D**: Percentage of HLA-DR-positive myofibers according to the degree of CD8+ T-cell infiltration. Comparison was done between patients with a CD8+ score null and patients with score equal to 1 or 2, ASM and DM patients having been pooled.

CD8+ T-cells were more abundant in ASM (Figure 4A-D). The percentage of HLA-DR-positive myofibers was positively correlated with the number of CD8+ cells (p < 0.05; Figure 4D). CD8+ cells were mainly observed in perifascicular endomysium and in perimysium, close to the border of fascicles, and colocalized with HLA-DR expression (Figure 4A-B). No correlation was found between HLA-DR expression and the number of CD3+, CD4+ or CD68+ cells (data not shown).

## Discussion

From present results, anti-synthetase myopathy (ASM) appears characterized by (i) a strong myofiber HLA-DR expression, electively localized in perifascicular areas, and (ii) complement activation with C5b-9 deposition at the surface of perifascicular myofibers. This peculiar immunopathological pattern has not been described so far, and appears strikingly distinct to that observed in DM, in which myofiber HLA-DR expression is only occasional or absent, and C5b-9 deposition mainly observed in endomysial capillaries. Our results reinforce the view that ASM corresponds to a unique entity resulting from a specific pathophysiological process.

Identifying new distinctive biomarkers for ASM will necessarily contribute to improve the diagnostic approach. The diagnosis of AS depends on the positive detection of detection of circulating antibodies directed towards anti-aminoacyl-tRNA synthetase molecules. However, only eight different ASAbs have been identified so far, that is few considering the number of amino-acids. Delineating a characteristic myopathological pattern for ASM could help to identify patients with “seronegative” anti synthetase syndrome and therefore prompt the identification of new antisynthetase antibodies.

Our results showed that similarities between ASM and DM also exist at the histopathological level. Indeed, both fragmentation of perimysial connective and perifascicular atrophy were found as frequent in ASM as in DM (79.3% vs 80.0%, NS: 44.4% vs 53.3%, respectively). HLA-ABC and NCAM were equally expressed in both conditions. Focal myofilament loss and microinfarctus were electively observed in DM and can represent distinctive features. However, the irregular presence of these features(40% and 33% in DM, respectively) dims their helpfulness. In this context, HLA-DR and C5b-9 proved to be more determining biomarkers for diagnostic delineation. Myofiber HLA-DR expression is clearly distinctive between ASM and DM. Distinguishing DM and ASM is not trivial since adult DM is significantly associated with cancer, as in our cohort. Interestingly none of our ASM patients had malignancy, a result in contrast with recent data showing an overall 12% frequency of cancer in patients with ASAbs [13]. Whether the immunopathological pattern we described in ASM may have a prognostic impact deserves further studies.

Only a few works previously addressed the issue of myofiber HLA-DR expression in muscle pathology [18,19,21,25,30]. Reports of positive myofiber HLA-DR expression mainly concerned patients classified as “polymyositis” (PM), but without indication about the association (or not) with anti-synthetase antibodies [19,25]. In these cases, HLA-DR-positive fibers were usually few and randomly distributed [19,25]. Interestingly, the lack of myofiber HLA-DR expression in DM was repeatedly observed [19,21,25], but never regarded as a potential distinctive biomarker. The marked myofiber HLA-DR expression and its perifascicular pattern we described in ASM have never been reported so far and indubitably constitute a new feature in myopathology.

The presence of extensive C5b-9 deposition at the surface of perifascicular myofibers in AS has not been previously reported to our knowledge. C5b-9, also termed membrane attack complex (MAC), is the cytolytic effector of complement system, produced after activation through either ‘classical’ antibody-dependent or alternative pathway. MAC formation in muscle pathology remains a puzzling phenomenon observed in various acquired and non-acquired conditions [1]. It is generally admitted that it does not reflect antibody-mediated immune attack [31,32]. Therefore, in ASM, sarcolemmal MAC deposits could reflect the presence of myofiber alterations in relation with the pathological process that also leads to HLA-DR expression. Complement activation may also contribute to myofiber necrosis and thus could constitute an amplifying factor of muscle injuries.

Unlike MHC class I expression, the expression of MHC class II molecules is most often restricted to antigen presenting cells (APCs) (macrophages, cells dendritic cells, B lymphocytes), at the notable exception of endothelial cells that constitutively express MHC class II in vivo [14,33]. MHC class II expression can be induced by IFNγ in various non-APCs, including mesenchymal stromal cells, fibroblasts and epithelial cells [19,34], and by hypoxia in cultured endothelial cells [33]. IFNγ is produced predominantly by natural killer (NK) and natural killer T (NKT) cells as part of the innate immune response, and by CD4 Th1 and CD8 cytotoxic T lymphocyte (CTL) effector T cells once antigen-specific immunity develops [35]. In accordance with these data, we found a quantitative correlation between HLA-DR expression andCD8 cells infiltrates (Figure 4). Moreover, CD8 cells were most often observed in close vicinity to HLA-DR expressing myofibers, in perifascicular endomysium or perimysium (Figure 4). HLA-DR expression is under the control of CIITA (Class II transactivator), a cytosolic protein itself controlled by the secretion of IFNγ [36]. In PM, HLA-DR expression was shown associated with that of IFNγ, Ii, CIITA and HLA-DM [19], and it seems sound to consider that HLA-DR expression reflects the presence of IFNγ in myofiber microenvironment. This view is supported by experimental data showing that myogenic cells respond to IFNγ by expressing HLA-DR. However, this effect of IFNγ seems restricted to proliferating and undifferentiated myoblasts, myotubes and innervated contracting muscle cells being unresponsive to IFNγ [20,26]. Whether mature myofiber may respond to IFNγ has not been documented to date, and mechanisms leading to myofiber HLA-DR expression still remain undetermined.

## Conclusion

In conclusion, our results confirm that ASM constitutes a specific type of primary inflammatory and dysimmune myopathies, in addition to PM/IBM, DM and AINM. In particular, ASM can be distinguished from other conditions by a strong myofiber MHC-II/HLA-DR expression, which should be henceforth considered as a key immunopathological biomarker for the diagnosis of IDM, in addition to MHC-I/HLA-ABC and C5b-9. HLA-DR expression suggests a role for type 2 IFNγ in ASM, in contrast with DM that relates to type 1 IFNα/β-mediated immune mechanism [31].

## Additional file

Additional file 1: Table S1. Primary antibodies used for immunohistochemical study.
