# Supplementary material for: Myofiber HLA-DR expression is a distinctive biomarker for antisynthetase-associated myopathy
Source: Acta Neuropathol Commun. 2014 Oct 23;2:154. doi: 10.1186/s40478-014-0154-2 (PMC4210467; doi:10.1186/s40478-014-0154-2)
Supplement: Additional file 1: Table S1. — Primary antibodies used for immunohistochemical study. [file 40478_2014_154_MOESM1_ESM.doc]

**Supplemental material: Table 1**

Primary antibodies used for immunohistochemical study

| Specificity | Clone | Brand | Dilution |
| --- | --- | --- | --- |
| HLA-ABC | W6/32 | Dako, Glostrup, Denmark | 1/4000 |
| HLA-DR | CR3/43 | Dako, Glostrup, Denmark | 1/2000 |
| MAC/C5b-9 | aE11 ;#ab55811 | Dako, Glostrup, Denmark | 1/50 |
| CD56/NCAM | #NCL-CD56-1B6 | Novocastra, Antony, France | 1/100 |
| CD68 | KP1 | Dako, Glostrup, Denmark | 1/300 |
| CD3 | F7.2.38 #A0452 | Dako, Glostrup, Denmark | 1/100 |
| CD4 | 4B12 #ncl-cd41f6 | Novocastra, Antony, France | 1/200 |
| CD8 | C8/144B #M7103 | Dako, Glostrup, Denmark | 1/400 |
| CD20 | L26 #M0755 | Dako, Glostrup, Denmark | 1/500 |
